# From G protein-coupled receptors to solute carriers - mass spectrometry informs dynamics, function and regulation

**DOI:** 10.1016/j.chempr.2024.02.022

**Published:** 2024-08-08

**Authors:** Haiping Tang, Carol V. Robinson

**Affiliations:** 1 Department of Chemistry, University of Oxford, Oxford, OX1 3TA, UK; 2 Kavli Institute for Nanoscience Discovery, Oxford, OX1 3QU, UK

## Abstract

Capturing the dynamics of G protein-coupled receptors (GPCRs) as they transduce extracellular signals into key physiological processes remains one of the most challenging research areas over the last two decades. Meanwhile more recent attention has turned to the action of solute carriers (SLCs) that comprise both facilitative transporters and secondary active transporters. Since GPCRs and SLCs play critical roles in health and disease, and thereby serve as major targets for drug discovery, much effort has focused on capturing their various conformational states using conventional structural biology techniques. Here we consider roles that mass spectrometry-based approaches play, particularly with respect to uncovering the impact of phosphorylation, defining ligands and revealing the action of lipids in modulating interactions, transport, and conformational dynamics.

## Introduction

The first native mass spectrometry (MS) of membrane protein complexes, in which interactions between cytoplasmic and membrane protein subunits were maintained, revealed the presence of posttranslational modification (PTM) and nucleotide binding^1^. These early results prompted notable milestones in terms of the mass and complexity of prokaryotic systems studied by native MS, including the 26 subunits of rotary ATPases^2^. While the obvious next step was to advance to receptors and transporters, specifically GPCRs and SLCs, this challenge was not straightforward. Parenthetically, both GPCRs and SLCs share common characteristics such that after detergent extraction from the lipid bilayer their activities and structures may be compromised^3^. Moreover, in the gas phase of the mass spectrometer, maintaining their folded structures and interactions is particularly challenging due to their dynamic structures. Therefore, in common with early attempts for GPCR structure determination, native MS relied heavily on thermally stabilising mutations, nanobody stabilisation, G-protein mimetics and truncated constructs as well as significant optimisation of detergent and lipid conditions^4^.

Considering first the properties of GPCRs their dynamic seven transmembrane helices endow them with a ready access for ligands. The diversity of this large family with 826 members means that GPCRs are able to respond to a variety of endogenous stimuli including light^3^, ions^4^, small organic compounds^5,6^, peptides^7^ and proteins^8^. GPCRs are targeted by approximately 35% of approved drugs^9^. This contrasts somewhat with the properties of SLCs which show high functional and structural variabilities and are increasingly acknowledged as an untapped source of potential new drug targets. SLCs represent the second-largest family of membrane proteins with around 456 members^10^, distributed in 65 subfamilies^11^. SLCs transport a wide range of solutes across both cellular and organellar membranes, including ions, sugars, lipids, amino acids, peptides, nucleotides, and other molecules^12,13^. Despite the fact that SLCs play pivotal roles in essential physiological functions, ~30% of SLCs are still functionally orphan with unknown substrates^14^. Fortuitously, there is growing interest in characterizing SLCs and systematically studying their therapeutic potential^15^. Since GPCRs and SLCs are both challenging targets, some degree of sequence truncation is often required to achieve improved expression, purification, and reconstitution yields^16^. As such many constructs used for structure determination have been devoid of unstructured loops and tails. One of the largest challenges for understanding the mechanism of many SLCs however is the identity of the driving force for transport and of the natural substrate for function.

Dramatic advances in single particle cryo-EM have made structure determination of GPCR complexes and SLCs, in all conformational states, considerably more straightforward than previous X-ray crystallography approaches^17,18^. The greater availability of structures, together with the growing confidence in protein structure prediction algorithms^19–21^, enables new situations to be considered. For example, how does the lipid environment or PTM status impact the structures of GPCRs and SLCs? Moreover, with structures in hand, uncovering the impact of ligand and lipid binding on the conformational dynamics of GPCRs and SLCs will enable greater understanding of their function.

To address these challenges, native MS and hydrogen-deuterium exchange MS (HDX-MS) are emerging as powerful complimentary approaches. Native MS, in which proteins and their ligands are sprayed from non-denaturing solutions, preserves noncovalent binding interactions upon transfer from solution to the gas phase^22^. Recording mass spectra of these interacting proteins therefore provides valuable information for structural biology such that subunit stoichiometry, binding partners and protein dynamics can be deduced. Of interest here is the stoichiometry and specificity of noncovalent membrane protein-lipid interactions and membrane protein-ligand complexes. In addition, HDX-MS also plays a pivotal role in deciphering unique structural information including secondary structure elements, protein dynamics and interaction interfaces. Unlike native MS, HDX-MS involves incubating proteins in a deuterium buffer to allow for the exchange with deuterium of labile hydrogens on protein backbone and side chains. Subsequently the exchange of deuterium by hydrogen in aqueous solution is then monitored as a mass change as a function of time. By comparing the deuterium uptake differences of proteins under different experimental conditions, structural dynamics can be assessed. Combining native MS to reveal ligand or protein binding events, with dynamics from HDX-MS, therefore provides a powerful combination to report on simultaneous binding and dynamics of membrane proteins under a variety of experimental conditions.

**Figure 1:**
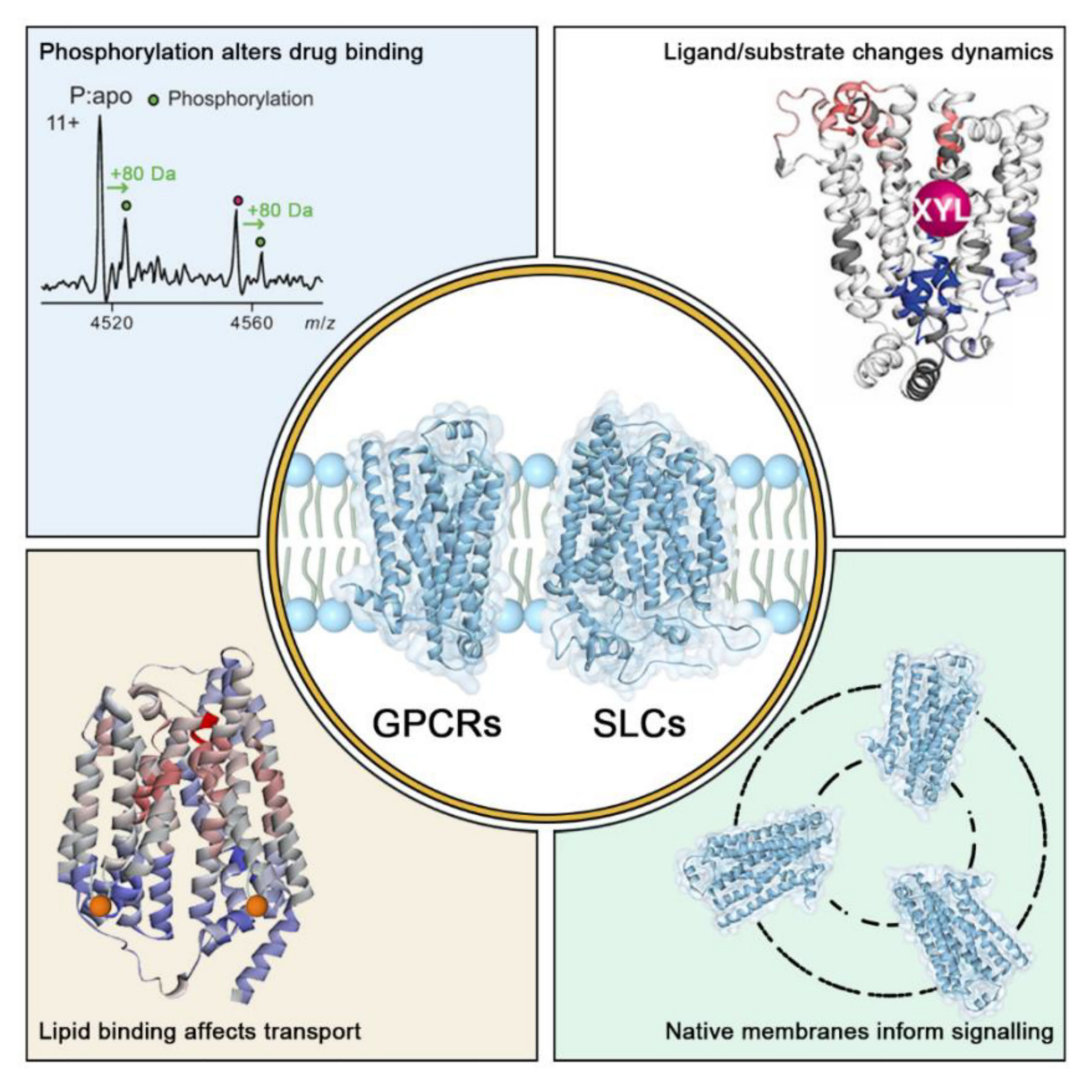
Overview of this perspective. The dynamic nature of GPCRs and SLCs, and their dependence on the membrane environment, render them challenging to study by established structural biology approaches alone. In this perspective we outline additional information that can be obtained from mass spectrometry approaches particularly with respect to the impact of phosphorylation on drug binding, the effect of ligands / substrates and lipid binding to the conformational dynamics of GPCRs and SLCs, and the ejection of receptors from native membranes to capture signal transduction.

In this perspective we describe the deduction of modifications and interactions that provide links to the function of GPCRs and SLCs. What follows is a series of examples in which native MS and HDX-MS combine to illustrate these insights. We include: the impact of phosphorylation on drug binding; the conformational dynamics of receptors and transporters reported in a time-resolved manner which can be difficult to extrapolate from static EM images but can be effectively recreated with multiple HDX time courses in the presence of different agonists, allosteric modulators, or critical lipids. We also review the most recent advances in which receptors are ejected directly from native membrane vesicles and look forward to new technologies that will render native MS ever more powerful (Figure 1). Our ultimate goal in the development of these native MS approaches is to provide as complete a picture as possible of the interactions of GPCRs and SLCs in their native eco-systems with the myriad of modifications, ligands and surrounding lipids that fine tune their regulation, signalling and function.

## Phosphorylation modulates drug binding and conformational dynamics

Phosphorylation is a relatively straightforward PTM to observe if native mass spectra are sufficiently well-resolved. A clear +80 Da peak can be confirmed through phosphatase treatment. The relative abundance of phosphorylated proteins is generally low *in vivo*^23^ and turnover is typically fast making this modification typically difficult to capture in most structural biology approaches. However, it is not simply the observation of the peak that is important but rather its consequences for drug binding or conformational dynamics.

**Figure 2:**
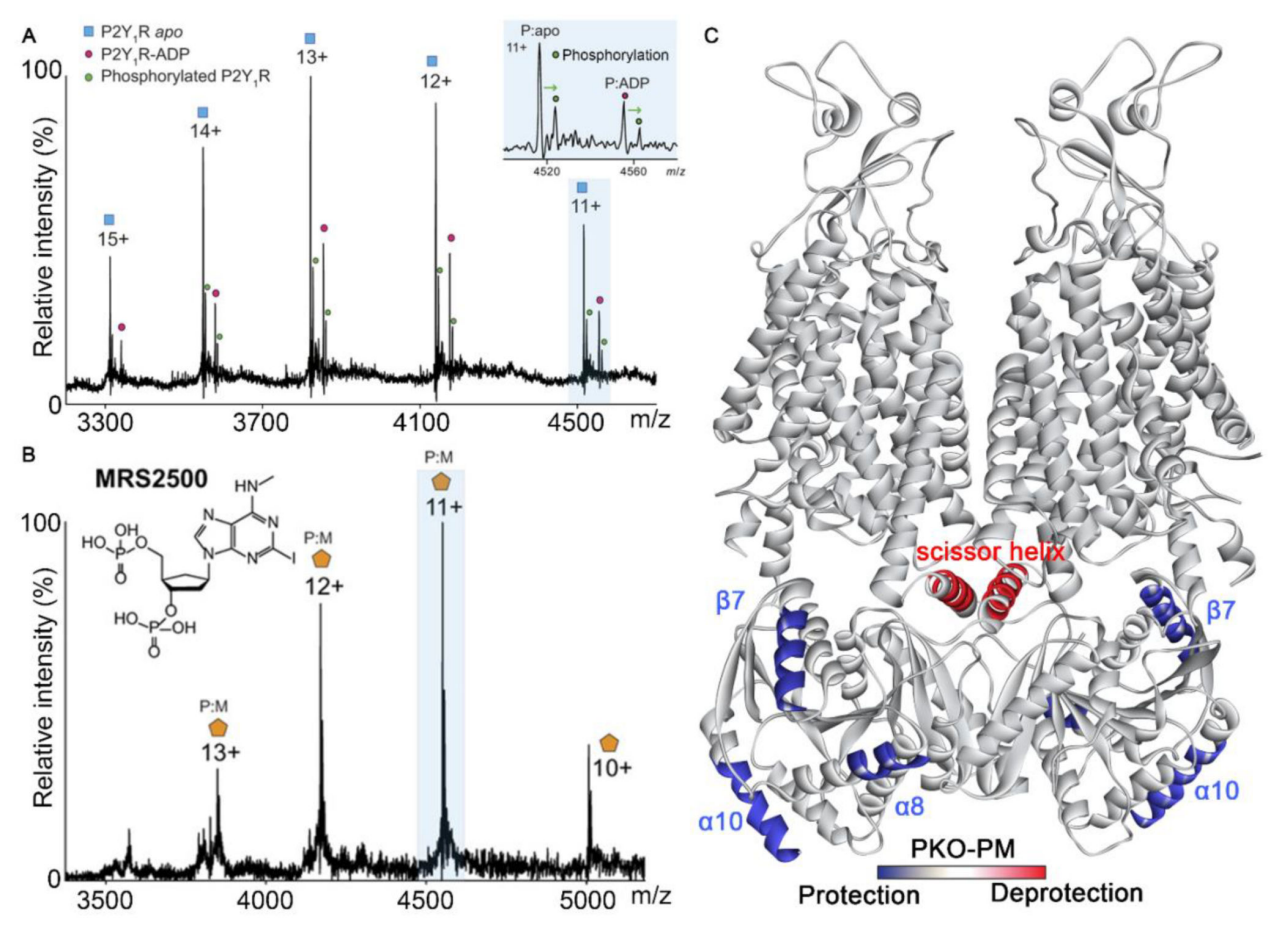
Drug binding of P2Y_1_R and conformational dynamics of KCC3b are impacted by phosphorylation (A) Native mass spectrum of P2Y_1_R (apo, blue square, charge states 11+ to 19+ are shown) with ADP binding (magenta circle) and phosphorylation (green circle). Expansion of the 11+ charge state (blue background) shows significant phosphorylation (green) of apo and ADP-bound forms. Reprinted from Yen et al.^31^ with permission. Copyright 2017, American Association for the Advancement of Science. (B) Native mass spectrum of P2Y_1_R purified in the presence of MRS2500 shows 100% binding to MRS2500, with no evidence for phosphorylation. Reprinted from Yen et al.^31^ with permission. Copyright 2017, American Association for the Advancement of Science. (C) Relative fractional uptake differences of KCC3b-PKO compared with KCC3b-PM mapped onto the structure of KCC3b-PM (PDB: 7AIO). Regions with increased or decreased deuterium uptake are coloured in red or blue respectively. Reprinted from Chi et al.^38^ with permission. Copyright 2021, EMBO Press.

One of the earliest examples of native MS of a GPCR reported the study of the human purinergic receptor (P2Y_1_R). P2Y_1_R is a receptor for adenosine 5’-triphosphate (ATP) and adenosine 5’-diphosphate (ADP)^24,25^ and is required for ADP-induced platelet aggregation^26^. Its inhibition results in a significant decrease of platelet aggregation^27^, which makes it as a key antithrombotic drug target^28^. MRS2500 is a potent antagonist of P2Y_1_R, which binds at the ATP binding site to inhibit ADP induced platelet aggregation and reduces arterial thrombosis^29,30^. P2Y_1_R protein, when captured in a folded state via native MS, revealed significant phosphorylation of the receptor (Figure 2A). Meanwhile, peaks with noncovalent binding of ADP were detected due to addition of ATP during the early stages of purification (Figure 2A). In addition, when P2Y_1_R proteins were purified in the presence of MRS2500, and subjected to native MS, 100% MRS2500 binding to P2Y_1_R was captured with no detectable ATP binding in-line with the competitive binding of this ligand (Figure 2B). Interestingly, no phosphorylated form of P2Y_1_R was observed in the presence of MRS2500, indicating that MRS2500 inhibits phosphorylation of P2Y_1_R. On the other hand, when MRS2500 was incubated with a mixture of unmodified and phosphorylated P2Y_1_R, MRS2500 was found to bind primarily to the non-phosphorylated form. This example highlights an allosteric effect of C-terminal phosphorylation which hinders access to the drug binding site^31^. Both the inhibition of phosphorylation, in the presence of the drug, and the effect of this PTM on drug binding would be difficult to observe using conventual structural biology approaches.

Turning to solute carriers, a growing number of structures have been determined as a consequence of technical breakthroughs in structure biology^32^. However, the structures that have been solved often represent static snapshots of the most stable states; conformations of transient states induced by substate binding and PTMs have been more difficult to capture. The potassium-coupled chloride transporters (KCCs) are a case in point. This SLC family plays a pivotal role in regulating intracellular chloride concentration and maintaining osmotic homeostasis^33^. Dysregulation of KCCs has been involved in various human diseases^34,35^. The transport activities of KCCs had been shown to be inhibited by phosphorylation^36,37^, but the molecular mechanism of this inhibition was unknown.

To shed light on this mechanism, HDX-MS was used to probe the conformational changes induced by phosphorylation of KCC3b. To assess the impact of phosphorylation, comparative HDX-MS was performed on a KCC3b phospho-knockout variant (KCC3b-PKO, which is constitutively active) and a phospho-mimetic variant (KCC3b-PM, which is phospho-inhibited). Regions with significant difference in deuterium uptake are in the outer lobe of the KCC3b C-terminal domain (β7, α8 and α10). The KCC3b-PKO variant exhibits decreased deuterium uptake in these regions when compared with KCC3b-PM variant (Figure 2C)^38^, suggesting decreased conformational dynamics in the KCC3b-PKO. Interestingly, increased deuterium uptake was observed in the scissor helix for KCC3b-PKO compared to KCC3b-PM (Figure 2C), a hinge region for the C-terminal domain movement. This result indicates that phosphorylation reduces the C-terminal domain movement of the KCC3b-PM variant thereby inhibiting its activity. Taken together, these studies highlight the capability of MS to interrogate the impact of PTMs on conformational dynamics.

## Ligand binding modulates conformational dynamics of GPCRs

**Figure 3:**
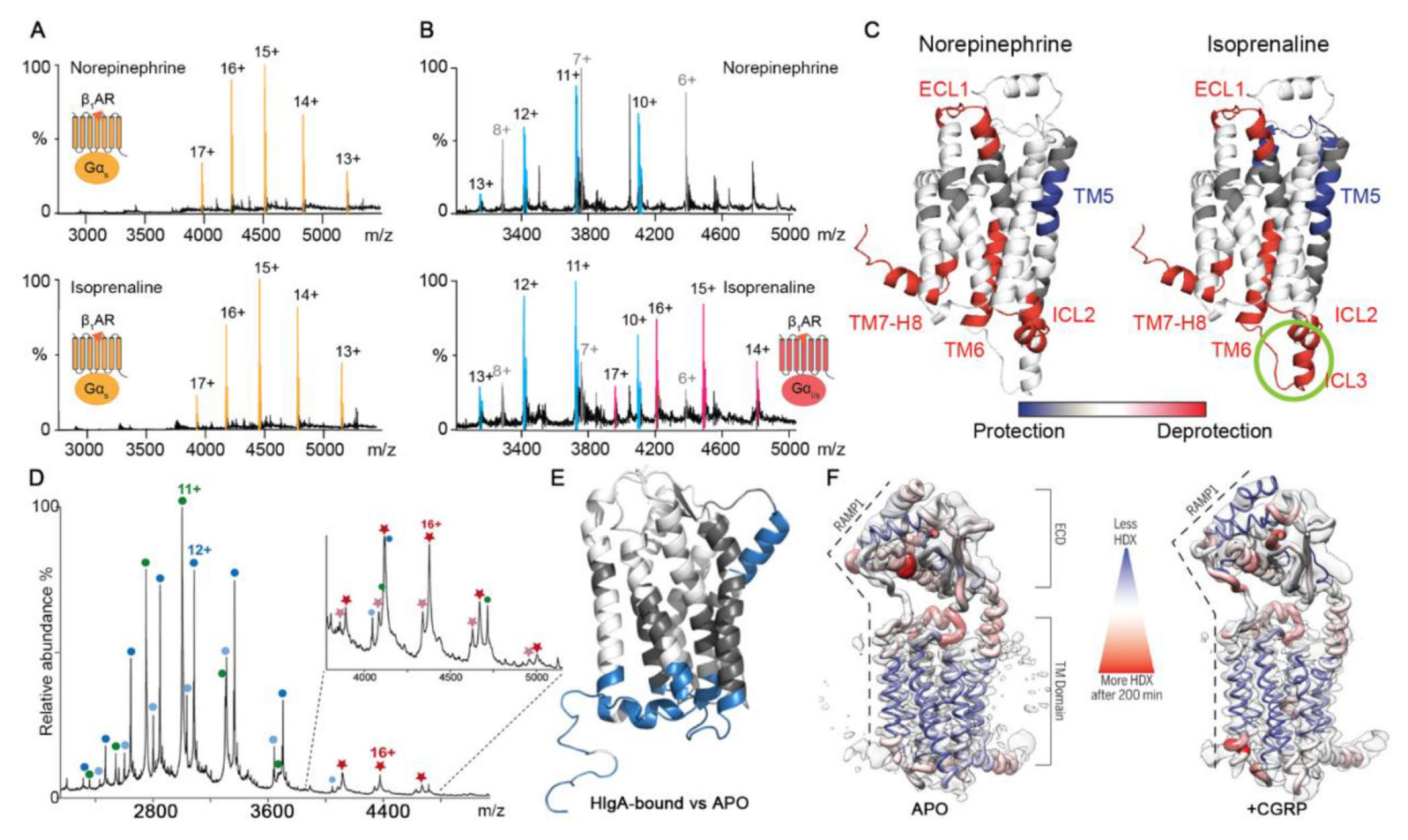
Monitoring complex formation and conformational dynamics of GPCRs using native MS and HDX-MS (A) Native mass spectra of tβ1AR complexed with mini-G_s_ in the presence of the agonists norepinephrine and isoprenaline. The peaks assigned to the receptor-mini-G_s_ complex are coloured orange. Reprinted from Yen et al.^39^ with permission. Copyright 2022, Nature Publishing Group. (B) Native mass spectra of tβ1AR-mini-G_i/s_ complexes formed in response to ligands norepinephrine and isoprenaline. The signals of the tβ1AR-mini-G_i/s_ complex, receptor monomer and mini-G_s_ are highlighted in magenta, blue and grey, respectively. Reprinted from Yen et al.^39^ with permission. Copyright 2022, Nature Publishing Group. (C) Different deuterium uptake pattern between agonist-bound and apo tβ1AR mapped onto the structure of tβ1AR (PDB: 2Y03). Regions with increased or decreased deuterium uptake compared to the apo state are coloured in red or blue respectively. The ICL3 motif is uniquely modulated by isoprenaline, highlighted by the green circle. Reprinted from Yen et al.^39^ with permission. Copyright 2022, Nature Publishing Group. (D) Native mass spectrum of a mixture of 2 mM HlgA and 5 mM ACKR1 treated with PNGaseF, showing the presence of monomeric HlgA (green circles), deglycosylated ACKR1 (blue circles), and partially hydrolyzed deglycosylated ACKR1 (light blue circles). Complexes formed between HlgA and both forms of deglycosylated ACKR1 are labelled with dark and clear red stars. Reprinted from Grison et al.^42^ with permission. Copyright 2021, National Academy of Sciences. (E) ACKR1 conformational changes induced upon binding to leukotoxin HlgA were probed by HDX-MS. Blue: protected regions; grey: regions with no statistically significant ΔHDX; and white: regions with no HDX data. Reprinted from Grison et al.^42^ with permission. Copyright 2021, National Academy of Sciences. (F) CGRP binding alters the dynamics of the CGRP receptor. HDX-MS data are illustrated in liquorice ribbon format, where the thickness and colour correspond to the extent of deuterium exchange. Reprinted from Josephs et al.^44^ with permission. Copyright 2021, American Association for the Advancement of Science.

Despite advanced insights from structural approaches, our understanding of ligands that modulate the structural dynamics of GPCRs to recruit transducer proteins remains incomplete. Before understanding the dynamics, it is necessary to capture the effects of ligand binding and G-protein coupling. Using a model system, of turkey β_1_-adrenergic receptor (tβ_1_AR) and mini-G_s_ with agonists, a native MS approach was developed^39^. For agonists norepinephrine, the natural ligand, and isoprenaline, complete receptor-mini-G_s_ complex formation was observed (Figure 3A). Since the β-adrenergic receptor can bind to G_i_ under certain stimulatory conditions^40,41^, mini-G_i/s_ was also tested to explore the stimulatory propensity of norepinephrine and isoprenaline. Interestingly, no tβ_1_AR-mini-G_i/s_ complex formation was detected for norepinephrine, whereas isoprenaline induced partial tβ_1_AR-mini-G_i/s_ complex formation (Figure 3B). These results suggest that isoprenaline was able to stimulate both mini-G_s_ and mini-G_i/s_ coupling of tβ_1_AR, whereas norepinephrine induced the preferential G_s_ signalling of tβ_1_AR. In addition, incubating tβ1AR with an equimolar solution of mini-G_s_ and mini-G_i/s_ revealed substantially more binding to mini-G_s_, indicative of biased G_s_ signalling of tβ1AR. These results highlight the sensitivity of this native MS approach to detect biased signalling through monitoring the interactions between receptors and G-proteins.

To understand the conformational changes associated with agonist-mediated biased signalling HDX-MS was performed. HDX results showed that both agonists norepinephrine and isoprenaline induced increased deuterium uptake in the extracellular loop 1 (ECL1), intracellular loop 2 (ICL2), and the intracellular ends of TM6 and TM7-H8, whereas decreased deuterium uptake was observed for TM5 (Figure 3C). Intriguingly isoprenaline, rather than norepinephrine, induced higher deuterium uptake in intracellular loop 3 (ICL3) (Figure 3C), indicating increased conformational dynamics thereby implying a role for this loop in promoting biased signalling pathways^39^.

Turning to an atypical chemokine receptor 1(ACKR1), a GPCR targeted by leukotoxins to promote bacteria immune evasion. Leukotoxin HlgA was shown in complex with ACKR1 via native MS (Figure 3D)^42^. HDX-MS was then used to demonstrate the effect of HlgA binding on the conformational dynamics of ACKR1. The results suggested that HlgA binding induced an allosteric conformational change of ACKR1 that leads to the protection of its intracellular part (Figure 3E). This region has proven critical for Gα binding^43^, indicating that leukotoxin binding leads to changes in the architecture of the receptor-G protein interaction. Overall, this finding brings molecular insights into the initial steps of leukotoxins targeting a host GPCR.

Beyond the small molecule ligands detailed above, neuropeptide induced conformational changes of the calcitonin gene-related peptide (CGRP) receptor (CGRPR) have been investigated in the presence of its neuropeptide ligand CGRP^44^. Compared to the apo protein, the CGRP-bound sample exhibited overall reduced deuterium exchange at the earlier time points and had increased deuterium uptake for the intracellular side of TM6 at the later time points (Figure 3F), suggesting CGRP binding allosterically modulates conformational changes of the intracellular face of CGRPR that might facilitate G protein binding.

With these examples we highlight how the classic allosteric properties of GPCRs^45^ via their conserved architecture conferring similarities to their functional conformations. Through divergent ligand binding at the extracellular side allosteric changes to the intercellular face of the receptor which can be captured by means of HDX-MS^46^. Meanwhile increased understanding of the impact of the intracellular loops and C-terminus upon G protein or arrestin binding, probed by HDX-MS, is providing further detail to the coupling mechanism^47,48^. The β_2_AR-G_s_ protein complex formation and G-protein activation were investigated by hydroxyl radical mediated protein foot printing with mass spectrometry (HRF-MS) and HDX-MS in a time-resolved manner from mini-second to minute timescale^49^. The results suggested that agonist-bound β_2_AR initially interacted with the C-terminal of the α5 helix of GDP-bound Gs followed by the interaction between ILC2 and Gs, a crucial step for GDP release^49^. This study provides unprecedented time resolution in monitoring the temporal sequence of G protein coupling events.

## Substrate and inhibitor binding induce distinct conformational changes of SLCs

**Figure 4:**
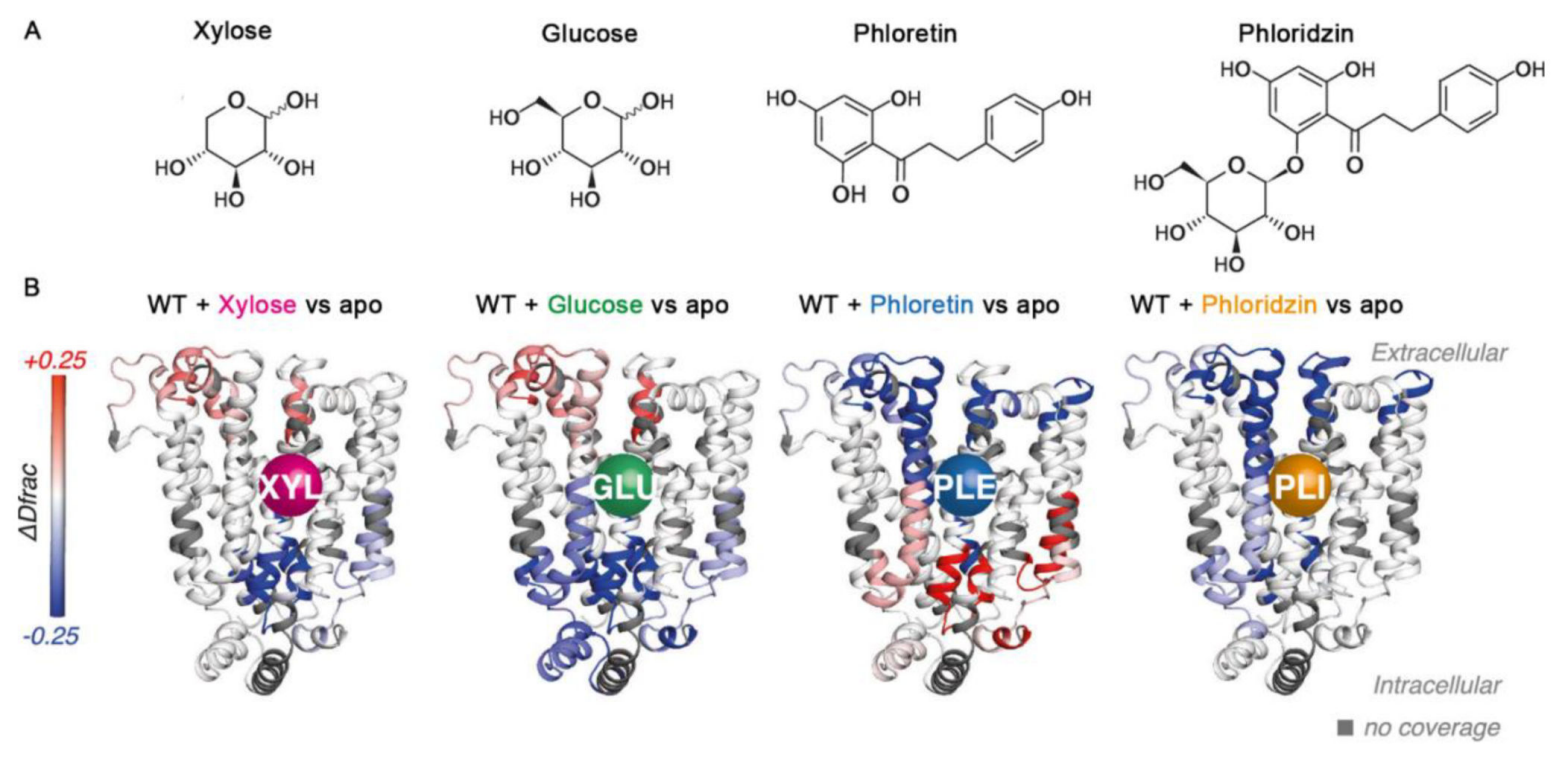
HDX-MS reveal conformational changes of WT XylE induced by ligands. (A) Structures of the ligands used in this study: xylose, glucose, phloretin, and phloridzin. (B) Differential HDX-MS uptake pattern between apo XylE and ligand-bound XylE mapped onto XylE structure (PDB:4GBY). Regions with increased or decreased deuterium uptake compared to the apo state are coloured in red or blue respectively. Regions with no coverage are coloured in dark grey. Reprinted from Jia et al.^54^ with permission. Copyright 2023, American Chemical Society.

Although it is widely accepted that SLCs alternate between open to the extracellular space (outward facing) and open to the cytoplasm (inward facing) to allow alternate exposure of their substrate binding sites^50^, the dynamical conformational changes of these transitions are difficult to deduce from structural snapshots. HDX-MS has been used to characterize the substrate and inhibitor induced conformational dynamics of XylE, an *Escherichia coli* homologue of human glucose transporters (Figure 4A). In the presence of substrate xylose and the endogenous inhibitor glucose, a decrease in deuterium uptake was observed on the intracellular side, with a concomitant increase on the extracellular side (Figure 4B). This is a typical HDX pattern for a transition towards an outward facing conformation. In contrast, the exogenous inhibitor phloretin^51^ induced an increased deuterium uptake on the intracellular side and a decreased deuterium uptake on the extracellular side, indicative of a typical transition to an inward facing conformation (Figure 4B). Intriguingly, the exogenous inhibitor phloridzin^52,53^ causes an overall decrease in deuterium uptake on both sides (Figure 4B). This patten is consistent with an occluded-like state^54^. Collectively, this work demonstrates the unique ability of HDX-MS to distinguish the conformational dynamics of substrate and diverse inhibitors with distinct modes of action.

## Lipid binding modulates G-protein coupling and conformational dynamics

**Figure 5:**
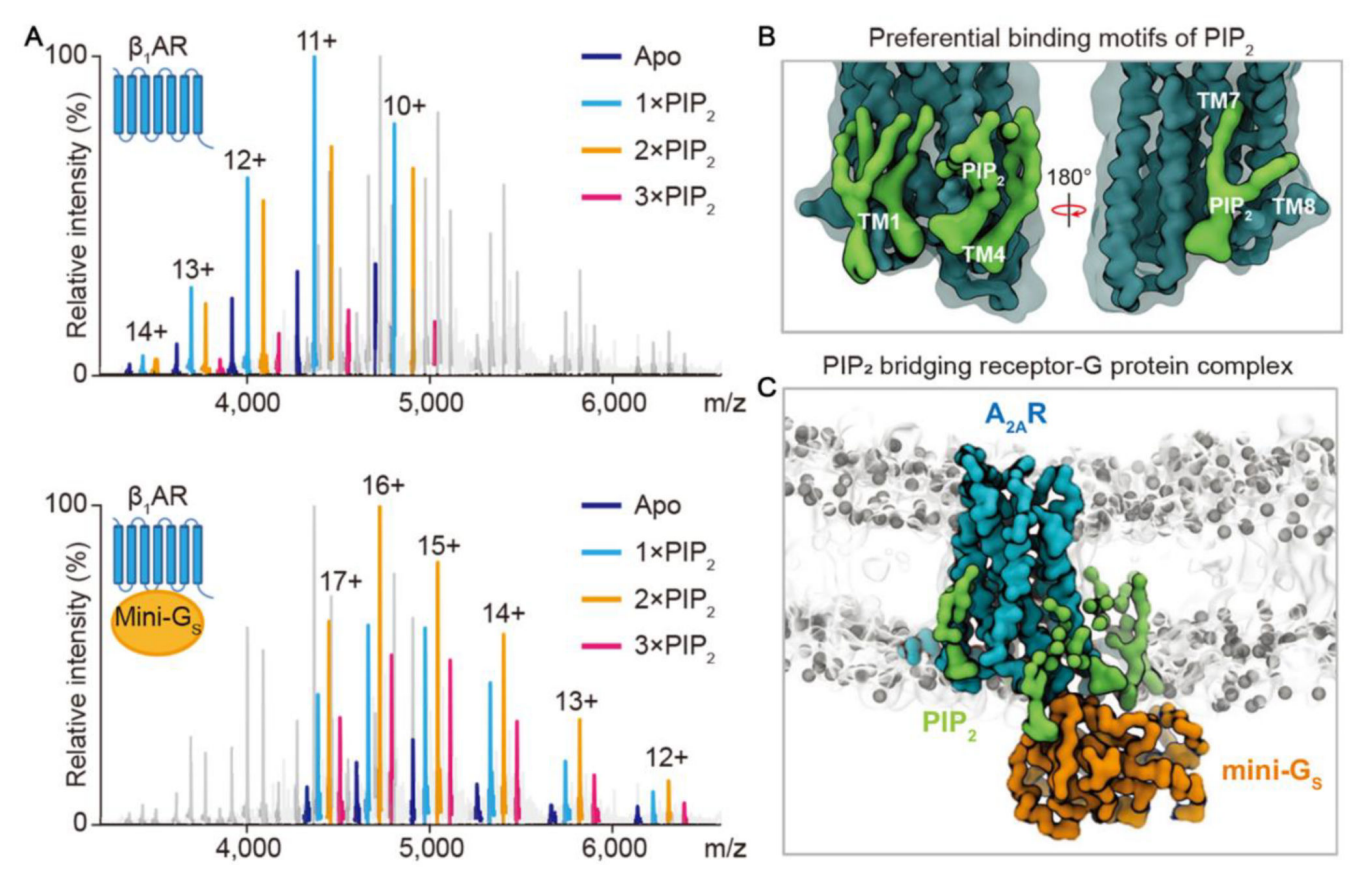
The effect of PIP_2_ on GPCR-mini-Gs coupling (A) Native mass spectrum of β_1_AR in complex with mini-G_s_ in the presence of PIP_2_ and the agonist isoprenaline. The peaks are highlighted differently to show β_1_AR lipid-bound state (top) and the higher relative intensity of the lipid-bound states for β_1_AR–mini-G_s_ (bottom). (B) Snapshot of molecular dynamics simulations for NTSR1 in the presence of PIP_2_ (green). (C) Snapshot of molecular dynamics simulations of mini-G_s_ (orange) and A_2A_R (blue) in the presence of PIP_2_ (green). Reprinted from Yen et al.^59^ with permission. Copyright 2018, Nature Publishing Group.

Given that GPCRs are integral membrane proteins that are embedded in lipid bilayers, the surrounding lipids will likely play substantial roles in regulating their functions^55^, stabilities^56^, oligomeric state^57^, and conformational dynamics^58^. Native MS has proved powerful in interrogating lipid-receptor interactions^59^. Preferential binding of phosphatidylinositol-4,5-bisphosphate (PIP_2_) for β_1_AR protein was identified by native MS (Figure 5A), implying that β_1_AR might contain binding sites for PIP_2_. Molecular dynamics simulations suggested preferential binding motifs of PIP_2_ and mutation of the suggested interacted residues was shown to attenuate PIP_2_ binding (Figure 5B).

To further investigate the effect of PIP_2_ on β_1_AR function, the extent of agonist-bound β_1_AR in complex with mini-G_s_ in the presence of PIP_2_ was examined by native MS. The results showed that the β_1_AR-mini-G_s_ complex formation was markedly enhanced when two or three PIP_2_ molecules are bound the complex (Figure 5C). Molecular dynamics simulations suggested that PIP_2_ may form bridging interactions at the interface between the receptor and mini-G_s_ (Figure 5D), implying that PIP_2_ molecules stabilize the active G-protein-bound state of the receptor^59^. In addition, PIPs was also reported to potentiate an active conformation of arrestin and stabilize GPCR-arrestin complexes^60^.

Interestingly, in contrast to β_1_AR, PIP_2_ was found to stabilize the inactive conformation of the glucagon receptor (GCGR)^61^. Initial native MS data showed a preferential binding of PIP_2_ to GCGR. Following molecular dynamics simulations PIP_2_ was suggested to have a higher affinity to the inactive conformation of the receptor and thereby stabilized its inactive conformation. Taken together, these results suggest that distinct signalling outcomes of GPCRs can be fine-tuned by membrane lipids.

## Lipids modulate conformational dynamics of SLCs

**Figure 6:**
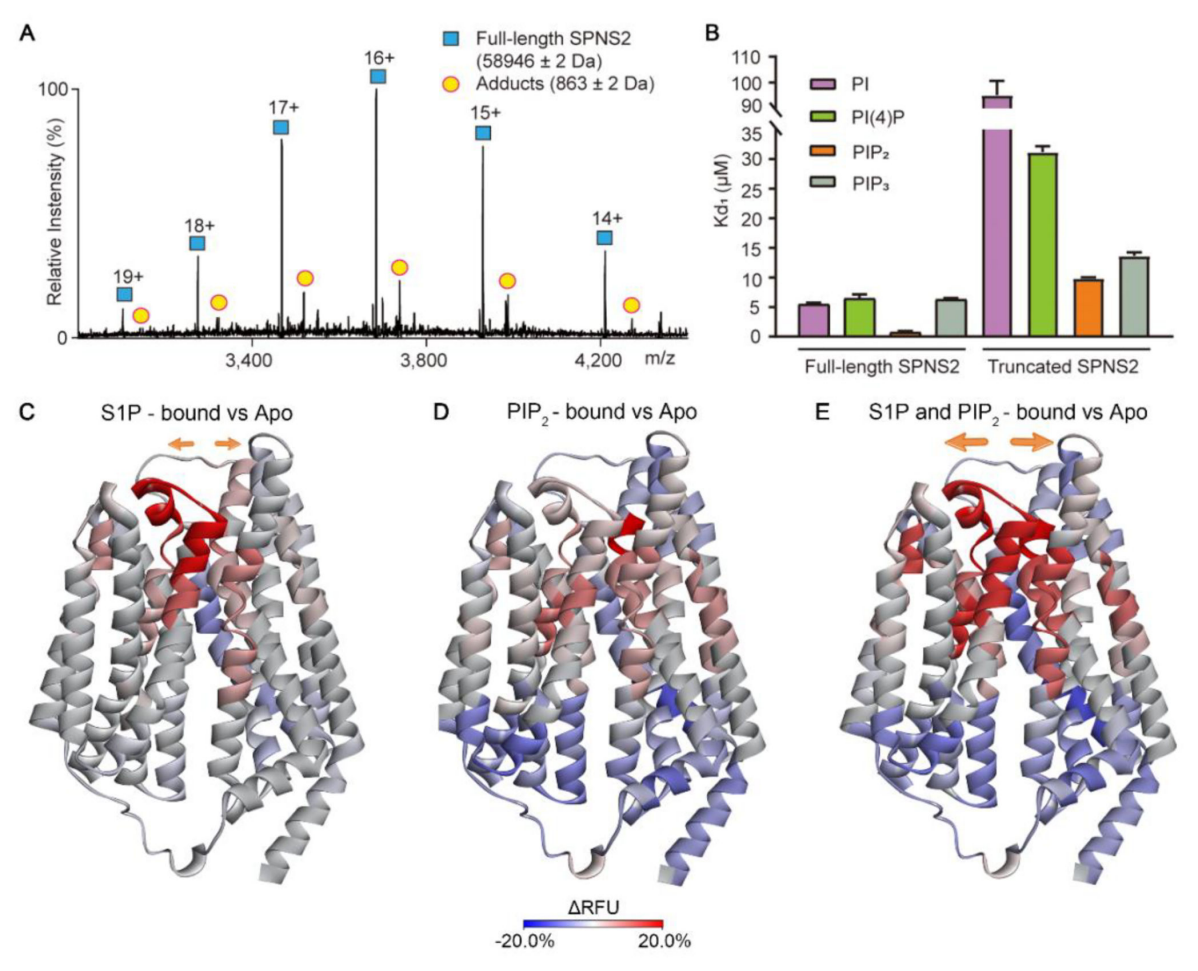
Binding preference of PI derivatives to full-length and truncated SPNS2 and conformational dynamics of SPNS2 in complex with S1P, PIP_2_, and both S1P and PIP_2_. (A) Native mass spectrum of full-length SPNS2 (blue, charge states 14+ to 19+ are shown) with PI adducts (yellow circles). (B) K_D1_ values for PI, PI(4)P, PIP_2_, and PIP_3_ binding to full-length and truncated SPNS2. Data are plotted as mean ± SD (n = 3). (C) Differential HDX-MS uptake pattern of S1P-bound SPNS2 compared with apo SPNS2 mapped onto the predicted model of SPNS2. (D) Differential HDX-MS uptake pattern of PIP_2_-bound SPNS2 compared with apo SPNS2 mapped onto the predicted model of SPNS2. (E) Differential HDX-MS uptake pattern of S1P and PIP_2_-bound SPNS2 compared with apo SPNS2 mapped onto the predicted model of SPNS2. Reprinted from Tang et al.^62^ with permission. Copyright 2023, Cell Press.

The impact of lipids on the functional regulations of SLCs has also been investigated recently^62^. Solute carrier spinster homolog 2 (SPNS2) is a sphingosine-1-phosphate (S1P) transporter in human, which exports S1P across cell membrane. Native MS analysis of SPNS2 protein purified from 293F cells showed that endogenous PI remained bound to SPNS2 after detergent extraction (Figure 6A). Since PI can be phosphorylated *in vivo*, the selectivity of SPNS2 for PI derivatives was investigated. Dissociation constants (K_D_) for PI derivatives were determined by native MS. Among the PI derivatives, PIP_2_ had the highest binding affinity to both full-length and N-terminally truncated SPNS2 (Figure 6B), indicating that PIP_2_ interacted preferentially with SPNS2. Intriguingly, N-terminally truncated SPNS2 showed a 10-fold weaker affinity for PIP_2_ compared to full-length SPNS2 (Figure 6B), suggesting that the N-terminus of SPNS2 is important for dictating its preference for PIP_2_.

Since this study was in advance of a high-resolution structure, an AlphaFold2 model was used to compare HDX-MS data. First HDX-MS was performed to validate the secondary structure elements predicted by the AlphaFold2 model. HDX was then used to further characterize the conformational changes of SPNS2 induced by PIP_2_ and its substrate S1P. Comparing the HDX-MS results of SPNS2 in the presence and absence of substrate S1P, an increase in deuterium uptake on the extracellular side of the SPSN2 was observed (Figure 6C), suggesting a dynamic opening of the extracellular gate of SPNS2 upon S1P binding. The analogous HDX experiment was carried out with and without PIP_2_. By calculating the differential relative fractional uptake (ΔRFU) with and without PIP_2_, a decreased deuterium uptake on the intracellular side of SPNS2, while increased deuteration on the extracellular side was deduced (Figure 6D). These differences suggest that PIP_2_ induces conformational changes of the intracellular side that allosterically regulate the extracellular side.

To further characterize the combined effect of S1P and PIP_2_ binding on the conformational dynamics of SPNS2, HDX-MS experiments were conducted for S1P-bound SPNS2 in the presence of PIP_2_. Comparison between the ternary complex and apo protein, revealed a decreased deuterium uptake on the intracellular side and a concomitant increased deuterium uptake on the extracellular side (Figure 6E). Moreover, the increased deuteration, induced by both S1P and PIP_2_, present simultaneously, is larger than the summed results of S1P and PIP_2_ individually^62^. Together these results suggest that PIP_2_ and S1P act synergistically to promote extracellular gate opening. In the case of SPNS2 the high confidence helical regions predicted in the Alphafold2 model are similar to the recently reported cryo-EM structure of SPNS2 with a root mean square deviation of 1.137 Å over 406 Cα^63^. More generally therefore this study highlights the mechanistic understanding of lipids and substrates on solute carrier’s transport can be deduced prior to high resolution structure determination using structure predictions.

## Interrogating GPCRs and SLCs from native membranes

A breakthrough in native MS has been to interrogate membrane protein complexes directly from their native membrane environment^64^. This approach does not require detergent or chemical intervention, but rather uses sonication or extrusion to generate lipid vesicles from native membranes. Following direct ejection of proteins from these vesicles into the mass spectrometer, intact membrane protein complexes and associated small molecules are retained^65^. Observation from inner mitochondrial membrane, revealed the solute carrier ANT1 in which a dimeric form could be captured bound to palmitate^64^.

**Figure 7:**
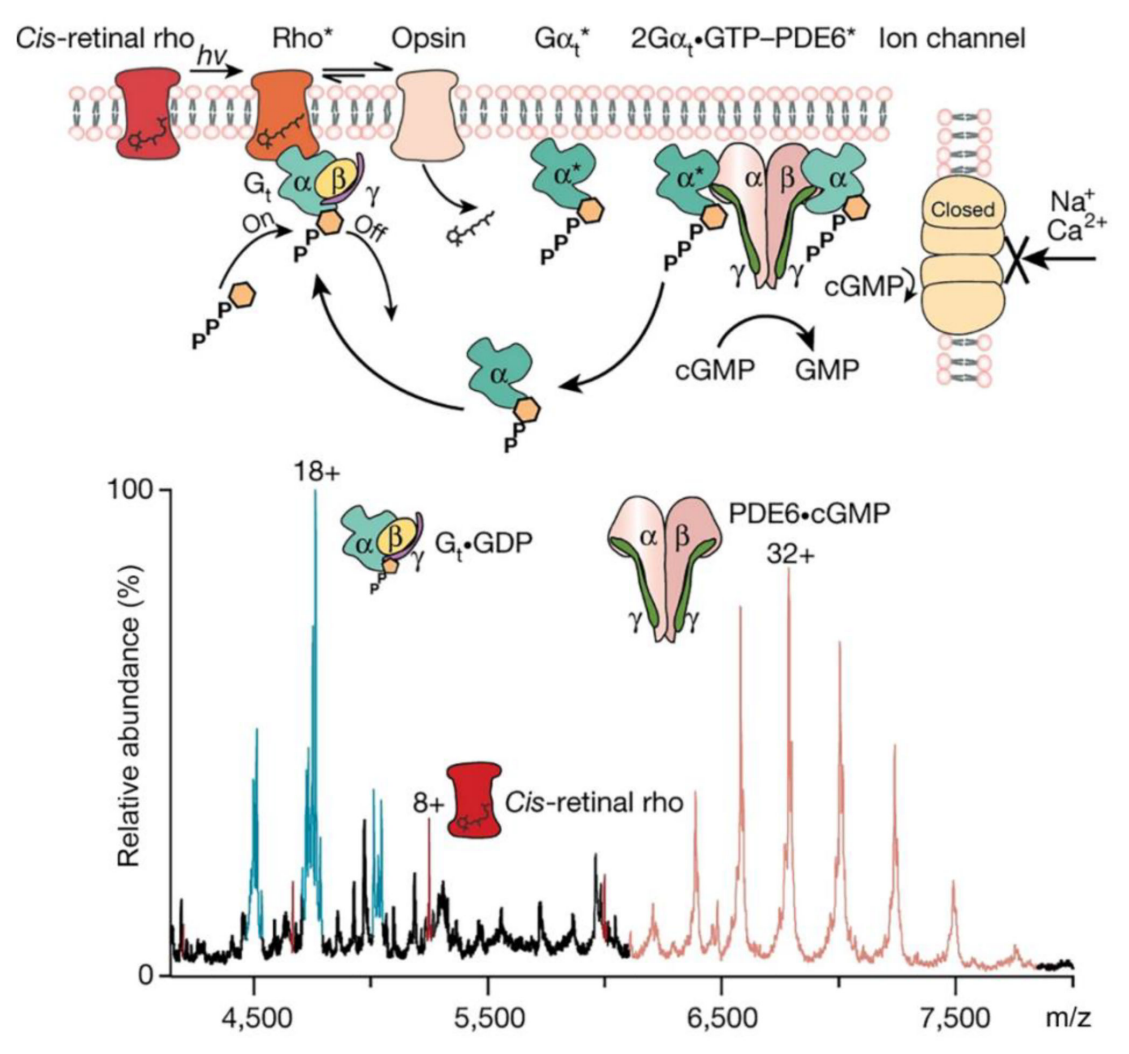
Light activation of rhodopsin in ROS disc membranes signals through G_t_ to release Gα_t_•GTP and activate PDE6 to hydrolyse cGMP. Schematic: Following absorption of a photon of light *hv*, 11-*cis*-retinal in rhodopsin (Rho) isomerizes to the all-*trans* isomer. The activated states of rhodropsin (rho*) engage with transducin (G_t_), consisting of Gα_t_•GDPβγ_t_, and exchange GDP for GTP. G_t_ dissociates to form Gα_t_•GTP and Gβγ_t_; loss of retinal from rho* leads to the formation of opsin. α-Subunits of G_t_ interact with the γ-subunits of the PDE6 enzyme, with γ-subunits undergoing a conformational change, relieving inhibition, and thereby activating PDE6 to cause hydrolysis of cGMP. Depletion of cGMP then closes the ion channel and the ‘dark current’ is terminated. Following the addition of a soluble fraction containing PDE6 and G_t_, all proteins along the signalling pathway were detected in native mass spectra (lower panel). The proteins were ejected intact as rhodopsin/opsin (red), trimeric G_t_•GDP (cyan) and tetrameric PDE6•cGMP (pink). Reprinted from Chen et al.^66^ with permission. Copyright 2022, Nature Publishing Group.

Further progress captured the signalling events of a wild-type class A GPCR rhodopsin, directly from bovine native disc membranes^66^. By controlling light exposure of the retina prior to analysis and then using pulses of light to promote formation of activated all-*trans*-retinylidene (rho*) from photoisomerization of 11-*cis*-retinylidene (*cis*-retinal rho). Supplementing the bovine disc membrane with GDP-bound heterotrimeric G protein transducin (G_t_ •GDP) and phosphodiesterase 6 (PDE6), when light activated rhodopsin interacted with G_t_ •GDP and exchanged GDP for GTP, which lead to dissociation of G_t_ and formation of Gα_t_ •GTP. Dissociated Gα_t_ •GTP then interact with PDE6, resulting in hydrolysis of cGMP (Figure 7). The whole process can be monitored by native MS due to the changes in mass. Importantly when ejected from this native membrane environment rhodopsin carries numerous PTMs including palmitoylation, glycosylation and phosphorylation and is ejected with a range of endogenous lipids that affect its function. As such these experiments move us closer to our overall goal - that of capturing wild-type receptor or transporter function in their native membrane eco-system.

In addition, the bovine ADP/ATP carrier was investigated by HDX-MS in its native mitochondrial membrane environment ^67^. The carrier within mitochondrial membranes was preincubated with inhibitor and then labelled in deuterated buffer. The resulting HDX-MS experiments were able to highlight significant differences in the conformational dynamics of bovine ADP/ATP carrier in the mitochondrial membrane from those in a detergent solution^68^. Similarly, A TonB-dependant vitamin B12 transporter BtuB was also interrogated by HDX-MS while still in its native *E.coli* outer membrane, highlighting structural changes induced by substrate B12 binding^69^. Furthermore, a protocol for *in vivo* HDX-MS in living *E. coli* cells, focusing on overexpressed BtuB was reported^70^. This *in vivo* labelling while in the outer membrane corroborated *in vitro* HDX-MS results, although a higher level of back exchange was observed. This study opens the possibility for future HDX-MS studies of protein dynamics in native cellular environments.

## Concluding remarks and future perspectives

The selected examples reviewed here were designed to showcase the ability of current mass spectrometry approaches to interrogate the roles of phosphorylation and the binding of ligands and lipids in modulating functions and structural dynamics of GPCRs and SLCs. While not exhaustive, they allow us to define the current state-of-the art.

Challenges remain however for charaterising these two important drug targets in the native states. For example, both GPCRs and SLCs often have extensive glycosylation. This modification dramatically increases the heterogeneity and complexity of native MS spectra making it difficult to define the binding of lipids or other endogenous ligands. Improvements in mass spectrometry instrumentation, with increased mass resolving power, and alternative methods of activating ions to release lipids and ligands are needed. A further challenge relates to the sequence coverage of GPCRs and SLCs HDX-MS analysis. Sequence coverage is dictated by the ability of pepsin to cleave the protein backbone, typically much lower than that obtained for soluble proteins, due to the paucity of fragments in transmembrane regions. Development of new protease combinations and optimization of the sample preparations are needed increase the sequence coverage. Still challenging are experiments designed to define unknown lipids and ligands within protein complexes. For such experiments multiple rounds of ion selection, activation, and fragmentation (MS^n^) are required^71^. Currently this approach is restricted to overexpressed solute carriers and GPCRs of relatively small size. However new instrumentation is imminent with increased mass range and alternative ways of activating ions. Specifically, instrumentation that enables infra-red activation offers the promise of not only controlled ejection from native membranes but also of performing multiple rounds of fragmentation of unknown proteins and cofactors^72,73^. Together with ever improving predictions of protein structure^20,21^, the detailed modifications and dynamics that can be added to these models will provide the missing links to their function.

A challenge that cannot be overcome is the physiological abundance of GPCRs and SLCs which are typically low. Consequently, most studies to date focus on recombinant proteins, which indeed accelerates our understanding of their function and regulatory mechanisms. However, the conditions of overexpressed recombinant proteins are likely different to those of the native cellular environment. Future efforts will be directed towards the interrogation of endogenous GPCRs and SLCs in their native membrane. Further developments in instrumentation with higher sensitivity, combined with advanced enrichment strategies and native separation strategies will bring this goal one step closer to reality. If this can be achieved informed drug discovery will result. Ultimately, we believe this development will enable comparison of GPCRs and SLCs, in healthy and diseased states, from their native ecosystem, to inform drug targeting and therapeutic intervention.

## References

[1] Barrera NP, Di Bartolo N, Booth PJ, Robinson CVJS (2008). Micelles protect membrane complexes from solution to vacuum.

[2] Zhou M, Morgner N, Barrera NP, Politis A, Isaacson SC, Matak-Vinković D, Murata T, Bernal RA, Stock D, Robinson CVJS (2011). Mass spectrometry of intact V-type ATPases reveals bound lipids and the effects of nucleotide binding.

[3] Nathans J, Hogness DSJC (1983). Isolation, sequence analysis, and intron-exon arrangement of the gene encoding bovine rhodopsin.

[4] Fredriksson R, Lagerström MC, Lundin L-G, Schiöth HBJMp (2003). The G-protein-coupled receptors in the human genome form five main families. Phylogenetic analysis, paralogon groups, and fingerprints.

[5] Dixon RA, Kobilka BK, Strader DJ, Benovic JL, Dohlman HG, Frielle T, Bolanowski MA, Bennett CD, Rands E, Diehl REJN (1986). Cloning of the gene and cDNA for mammalian β-adrenergic receptor and homology with rhodopsin.

[6] Kobilka BK, MacGregor C, Daniel K, Kobilka T, Caron M, Lefkowitz RJJoBC (1987). Functional activity and regulation of human beta 2-adrenergic receptors expressed in Xenopus oocytes.

[7] Masu Y, Nakayama K, Tamaki H, Harada Y, Kuno M, Nakanishi SJN (1987). cDNA eloping of bovine substance-K receptor through oocyte expression system.

[8] Parmentier M, Libert F, Maenhaut C, Lefort A, Gérard C, Perret J, Van Sande J, Dumont JE, Vassart GJS (1989). Molecular cloning of the thyrotropin receptor.

[9] Sriram K, Insel PAJMp (2018). G protein-coupled receptors as targets for approved drugs: how many targets and how many drugs?.

[10] Cesar-Razquin A, Snijder B, Frappier-Brinton T, Isserlin R, Gyimesi G, Bai X, Reithmeier RA, Hepworth D, Hediger MA, Edwards AMJC (2015). A call for systematic research on solute carriers.

[11] Bai X, Moraes TF, Reithmeier RAJMmb (2017). Structural biology of solute carrier (SLC) membrane transport proteins.

[12] Wang WW, Gallo L, Jadhav A, Hawkins R, Parker CGJJomc (2019). The druggability of solute carriers.

[13] Lin L, Yee SW, Kim RB, Giacomini KMJNrDd (2015). SLC transporters as therapeutic targets: emerging opportunities.

[14] Meixner E, Goldmann U, Sedlyarov V, Scorzoni S, Rebsamen M, Girardi E, Superti-Furga GJMSB (2020). A substrate-based ontology for human solute carriers.

[15] Dvorak V, Superti-Furga GJEOoDD (2023). Structural and functional annotation of solute carrier transporters: implication for drug discovery.

[16] Raturi S, Li H, Chang Y-N, Scacioc A, Bohstedt T, Fernandez-Cid A, Evans A, Abrusci P, Balakrishnan A, Pascoa TCJJoVE (2023). High-throughput expression and purification of human solute carriers for structural and biochemical studies.

[17] Gusach A, García-Nafría J, Tate CGJCOiSB (2023). New insights into GPCR coupling and dimerisation from cryo-EM structures.

[18] Baril SA, Gose T, Schuetz JDJDM, Disposition (2023). How Cryo-EM Has Expanded Our Understanding of Membrane Transporters.

[19] Jumper J, Evans R, Pritzel A, Green T, Figurnov M, Ronneberger O, Tunyasuvunakool K, Bates R, Žídek A, Potapenko AJN (2021). Highly accurate protein structure prediction with AlphaFold.

[20] Baek M, DiMaio F, Anishchenko I, Dauparas J, Ovchinnikov S, Lee GR, Wang J, Cong Q, Kinch LN, Schaeffer RDJS (2021). Accurate prediction of protein structures and interactions using a three-track neural network.

[21] Varadi M, Anyango S, Deshpande M, Nair S, Natassia C, Yordanova G, Yuan D, Stroe O, Wood G, Laydon AJNar (2022). AlphaFold Protein Structure Database: massively expanding the structural coverage of protein-sequence space with high-accuracy models.

[22] Leney AC, Heck AJJJotASfMS (2016). Native mass spectrometry: what is in the name?.

[23] Martenson R, Law M, Deibler GJJoBC (1983). Identification of multiple in vivo phosphorylation sites in rabbit myelin basic protein.

[24] Ayyanathan K, Webbs TE, Sandhu AK, Athwal RS, Barnard EA, Kunapuli SPJB, communications, b.r (1996). Cloning and chromosomal localization of the human P2Y1 purinoceptor.

[25] Schachter JB, Li Q, Boyer JL, Nicholas RA, Harden TKJBjop (1996). Second messenger cascade specificity and pharmacological selectivity of the human P2Y1-purinoceptor.

[26] Hechler B, Eckly A, Ohlmann P, Cazenave JP, Gachet CJBjoh (1998). The P2Y1 receptor, necessary but not sufficient to support full ADP-induced platelet aggregation, is not the target of the drug clopidogrel.

[27] Jin J, Kunapuli SPJPotNAoS (1998). Coactivation of two different G protein-coupled receptors is essential for ADP-induced platelet aggregation.

[28] Gachet CJT, haemostasis (2008). P2 receptors, platelet function and pharmacological implications.

[29] Hechler B, Nonne C, Roh EJ, Cattaneo M, Cazenave J-P, Lanza F, Jacobson KA, Gachet CJJoP, Therapeutics E (2006). MRS2500 [2-iodo-N6-methyl-(N)-methanocarba-2′-deoxyadenosine-3′, 5′-bisphosphate], a potent, selective, and stable antagonist of the platelet P2Y1 receptor with strong antithrombotic activity in mice.

[30] Wong PC, Watson C, Crain EJJJot, thrombolysis (2016). The P2Y 1 receptor antagonist MRS2500 prevents carotid artery thrombosis in cynomolgus monkeys.

[31] Yen H-Y, Hopper JT, Liko I, Allison TM, Zhu Y, Wang D, Stegmann M, Mohammed S, Wu B, Robinson CVJSA (2017). Ligand binding to a G protein–coupled receptor captured in a mass spectrometer.

[32] Drew D, North RA, Nagarathinam K, Tanabe MJCr (2021). Structures and general transport mechanisms by the major facilitator superfamily (MFS).

[33] Lauf PK, Bauer J, Adragna NC, Fujise H, Zade-Oppen A, Ryu K, Delpire EJAJoP-CP (1992). Erythrocyte K-Cl cotransport: properties and regulation.

[34] Kahle KT, Staley KJ, Nahed BV, Gamba G, Hebert SC, Lifton RP, Mount DBJNcpN (2008). Roles of the cation–chloride cotransporters in neurological disease.

[35] Kahle KT, Merner ND, Friedel P, Silayeva L, Liang B, Khanna A, Shang Y, Lachance-Touchette P, Bourassa C, Levert AJEr (2014). Genetically encoded impairment of neuronal KCC 2 cotransporter function in human idiopathic generalized epilepsy.

[36] Jennings ML, al-Rohil NJTJogp (1990). Kinetics of activation and inactivation of swelling-stimulated K+/Cl-transport. The volume-sensitive parameter is the rate constant for inactivation.

[37] de Los Heros P, Alessi DR, Gourlay R, Campbell DG, Deak M, Macartney TJ, Kahle KT, Zhang JJBJ (2014). The WNK-regulated SPAK/OSR1 kinases directly phosphorylate and inhibit the K+–Cl− co-transporters.

[38] Chi G, Ebenhoch R, Man H, Tang H, Tremblay LE, Reggiano G, Qiu X, Bohstedt T, Liko I, Almeida FGJTEj (2021). Phospho-regulation, nucleotide binding and ion access control in potassium-chloride cotransporters.

[39] Yen H-Y, Liko I, Song W, Kapoor P, Almeida F, Toporowska J, Gherbi K, Hopper JT, Charlton SJ, Politis AJNc (2022). Mass spectrometry captures biased signalling and allosteric modulation of a G-protein-coupled receptor.

[40] Daaka Y, Luttrell LM, Lefkowitz RJJN (1997). Switching of the coupling of the β2-adrenergic receptor to different G proteins by protein kinase A.

[41] Lefkowitz RJ, Pierce KL, Luttrell LMJMp (2002). Dancing with different partners: protein kinase a phosphorylation of seven membrane-spanning receptors regulates their G protein-coupling specificity.

[42] Grison CM, Lambey P, Jeannot S, Del Nero E, Fontanel S, Peysson F, Heuninck J, Sounier R, Durroux T, Leyrat CJPotNAoS (2021). Molecular insights into mechanisms of GPCR hijacking by Staphylococcus aureus.

[43] Mahoney JP, Sunahara RKJCoisb (2016). Mechanistic insights into GPCR–G protein interactions.

[44] Josephs TM, Belousoff MJ, Liang Y-L, Piper SJ, Cao J, Garama DJ, Leach K, Gregory KJ, Christopoulos A, Hay DLJS (2021). Structure and dynamics of the CGRP receptor in apo and peptide-bound forms.

[45] Weis WI, Kobilka BKJArob (2018). The molecular basis of G protein–coupled receptor activation.

[46] Zhang X, Chien EY, Chalmers MJ, Pascal BD, Gatchalian J, Stevens RC, Griffin PRJAc (2010). Dynamics of the β2-adrenergic G-protein coupled receptor revealed by hydrogen–deuterium exchange.

[47] Chung KY, Rasmussen SG, Liu T, Li S, DeVree BT, Chae PS, Calinski D, Kobilka BK, Woods VL, Sunahara RKJN (2011). β2 adrenergic receptor-induced conformational changes in the heterotrimeric G protein Gs.

[48] Shukla AK, Westfield GH, Xiao K, Reis RI, Huang L-Y, Tripathi-Shukla P, Qian J, Li S, Blanc A, Oleskie ANJN (2014). Visualization of arrestin recruitment by a G-protein-coupled receptor.

[49] Du Y, Duc NM, Rasmussen SG, Hilger D, Kubiak X, Wang L, Bohon J, Kim HR, Wegrecki M, Asuru AJC (2019). Assembly of a GPCR-G protein complex.

[50] Huang Y, Lemieux MJ, Song J, Auer M, Wang D-NJS (2003). Structure and mechanism of the glycerol-3-phosphate transporter from Escherichia coli.

[51] Krupka RJTJomb (1985). Asymmetrical binding of phloretin to the glucose transport system of human erythrocytes.

[52] Nelson J, Falk RJAr (1993). The efficacy of phloridzin and phloretin on tumor cell growth.

[53] Chan SS, Lotspeich WDJAJoP-LC (1962). Comparative effects of phlorizin and phloretin on glucose transport in the cat kidney.

[54] Jia R, Bradshaw RT, Calvaresi V, Politis AJJotACS (2023). Integrating Hydrogen Deuterium Exchange–Mass Spectrometry with Molecular Simulations Enables Quantification of the Conformational Populations of the Sugar Transporter XylE.

[55] Thakur N, Ray AP, Sharp L, Jin B, Duong A, Pour NG, Obeng S, Wijesekara AV, Gao Z-G, McCurdy CRJNC (2023). Anionic phospholipids control mechanisms of GPCR-G protein recognition.

[56] Oates J, Faust B, Attrill H, Harding P, Orwick M, Watts AJBeBA-B (2012). The role of cholesterol on the activity and stability of neurotensin receptor 1.

[57] Patil DN, Singh S, Laboute T, Strutzenberg TS, Qiu X, Wu D, Novick SJ, Robinson CV, Griffin PR, Hunt JFJS (2022). Cryo-EM structure of human GPR158 receptor coupled to the RGS7-Gβ5 signaling complex.

[58] Dawaliby R, Trubbia C, Delporte C, Masureel M, Van Antwerpen P, Kobilka BK, Govaerts CJNcb (2016). Allosteric regulation of G protein–coupled receptor activity by phospholipids.

[59] Yen H-Y, Hoi KK, Liko I, Hedger G, Horrell MR, Song W, Wu D, Heine P, Warne T, Lee YJN (2018). PtdIns (4, 5) P2 stabilizes active states of GPCRs and enhances selectivity of G-protein coupling.

[60] Janetzko J, Kise R, Barsi-Rhyne B, Siepe DH, Heydenreich FM, Kawakami K, Masureel M, Maeda S, Garcia KC, von Zastrow MJC (2022). Membrane phosphoinositides regulate GPCR-β-arrestin complex assembly and dynamics.

[61] Kjølbye LR, Sørensen L, Yan J, Berglund NA, Ferkinghoff-Borg J, Robinson CV, Schiøtt BJJoCI, Modeling (2022). Lipid modulation of a class B GPCR: elucidating the modulatory role of PI (4, 5) P2 Lipids.

[62] Tang H, Li H, Prakaash D, Pedebos C, Qiu X, Sauer DB, Khalid S, Duerr K, Robinson CVJMC (2023). The solute carrier SPNS2 recruits PI (4, 5) P2 to synergistically regulate transport of sphingosine-1-phosphate.

[63] Duan Y, Leong NC, Zhao J, Zhang Y, Nguyen DT, Ha HT, Wang N, Xia R, Xu Z, Ma ZJCR (2023). Structural basis of Sphingosine-1-phosphate transport via human SPNS2.

[64] Chorev DS, Baker LA, Wu D, Beilsten-Edmands V, Rouse SL, Zeev-Ben-Mordehai T, Jiko C, Samsudin F, Gerle C, Khalid SJS (2018). Protein assemblies ejected directly from native membranes yield complexes for mass spectrometry.

[65] Chorev DS, Tang H, Rouse SL, Bolla JR, von Kügelgen A, Baker LA, Wu D, Gault J, Gruenewald K, Bharat TAJNp (2020). The use of sonicated lipid vesicles for mass spectrometry of membrane protein complexes.

[66] Chen S, Getter T, Salom D, Wu D, Quetschlich D, Chorev DS, Palczewski K, Robinson CVJN (2022). Capturing a rhodopsin receptor signalling cascade across a native membrane.

[67] Rey M, Forest E, Pelosi LJB (2012). Exploring the conformational dynamics of the bovine ADP/ATP carrier in mitochondria.

[68] Rey M, Man P, Clémençon B, Trézéguet V, Brandolin G, Forest E, Pelosi LJJoBC (2010). Conformational dynamics of the bovine mitochondrial ADP/ATP carrier isoform 1 revealed by hydrogen/deuterium exchange coupled to mass spectrometry.

[69] Zmyslowski AM, Baxa MC, Gagnon IA, Sosnick TRJPotNAoS (2022). HDX-MS performed on BtuB in E. coli outer membranes delineates the luminal domain’s allostery and unfolding upon B12 and TonB binding.

[70] Lin X, Zmyslowski AM, Gagnon IA, Nakamoto RK, Sosnick TRJPS (2022). Development of in vivo HDX-MS with applications to a TonB-dependent transporter and other proteins.

[71] Gault J, Liko I, Landreh M, Shutin D, Bolla JR, Jefferies D, Agasid M, Yen H-Y, Ladds MJ, Lane DPJNm (2020). Combining native and ‘omics’ mass spectrometry to identify endogenous ligands bound to membrane proteins.

[72] Lutomski CA, El-Baba TJ, Hinkle JD, Liko I, Bennett JL, Kalmankar NV, Dolan A, Kirschbaum C, Greis K, Urner LHJACIE (2023). Infrared Multiphoton Dissociation Enables Top-Down Characterization of Membrane Protein Complexes and G Protein-Coupled Receptors.

[73] Juliano BR, Keating JW, Ruotolo BTJAC (2023). Infrared Photoactivation Enables Improved Native Top-Down Mass Spectrometry of Transmembrane Proteins.

